# Appropriate threshold levels of cardiac beat-to-beat variation in semi-automatic analysis of equine ECG recordings

**DOI:** 10.1186/s12917-016-0894-2

**Published:** 2016-11-28

**Authors:** Mette Flethøj, Jørgen K. Kanters, Philip J. Pedersen, Maria M. Haugaard, Helena Carstensen, Lisbeth H. Olsen, Rikke Buhl

**Affiliations:** 1 Department of Large Animal Sciences, Faculty of Health and Medical Sciences, University of Copenhagen, Hoejbakkegaard Allé 5, 2630 Taastrup, Denmark; 2 Laboratory of Experimental Cardiology, Department of Biomedical Sciences, Faculty of Health and Medical Sciences, University of Copenhagen, Blegdamsvej 3B, 2200 Copenhagen N, Denmark; 3 Department of Cardiology, Herlev & Gentofte University Hospitals, Copenhagen, Denmark; 4 Department of Veterinary Clinical and Animal Sciences, Faculty of Health and Medical Sciences, University of Copenhagen, Dyrlaegevej 100, 1870 Frederiksberg C, Denmark; 5 Department of Veterinary Disease Biology, Faculty of Health and Medical Sciences, University of Copenhagen, Groennegaardsvej 15, 1870 Frederiksberg C, Denmark

**Keywords:** ECG analysis, Heart rate variability, Arrhythmias, Endurance horses

## Abstract

**Background:**

Although premature beats are a matter of concern in horses, the interpretation of equine ECG recordings is complicated by a lack of standardized analysis criteria and a limited knowledge of the normal beat-to-beat variation of equine cardiac rhythm. The purpose of this study was to determine the appropriate threshold levels of maximum acceptable deviation of RR intervals in equine ECG analysis, and to evaluate a novel two-step timing algorithm by quantifying the frequency of arrhythmias in a cohort of healthy adult endurance horses.

**Results:**

Beat-to-beat variation differed considerably with heart rate (HR), and an adaptable model consisting of three different HR ranges with separate threshold levels of maximum acceptable RR deviation was consequently defined. For resting HRs <60 beats/min (bpm) the threshold level of RR deviation was set at 20%, for HRs in the intermediate range between 60 and 100 bpm the threshold was 10%, and for exercising HRs >100 bpm, the threshold level was 4%. Supraventricular premature beats represented the most prevalent arrhythmia category with varying frequencies in seven horses at rest (median 7, range 2–86) and six horses during exercise (median 2, range 1–24).

**Conclusions:**

Beat-to-beat variation of equine cardiac rhythm varies according to HR, and threshold levels in equine ECG analysis should be adjusted accordingly. Standardization of the analysis criteria will enable comparisons of studies and follow-up examinations of patients. A small number of supraventricular premature beats appears to be a normal finding in endurance horses. Further studies are required to validate the findings and determine the clinical significance of premature beats in horses.

## Background

Electrocardiography (ECG) is a valuable tool for the evaluation of cardiac arrhythmias in horses, yet analysis of the equine ECG is complicated by a lack of standardized criteria. Furthermore, the clinical interpretation of analysis results is challenged by limited knowledge of the clinical significance of various arrhythmias [1]. Both supraventricular and ventricular premature beats have raised concerns in equine cardiology as potential initiators of atrial fibrillation [2] and fatal ventricular arrhythmias [3]. In recent years, studies have therefore investigated the occurrence of premature beats in healthy horses [4–8], horses with poor performance [9, 10], and in both clinical [11, 12] and experimental settings [13]. These studies provide valuable information on the *prevalence* of premature beats, although a comparison of the results is problematic due to different study designs. The use of varying analysis criteria and ECG recordings of different duration impedes general conclusions about the *frequency* of premature beats in individual horses from the published studies. At what number, and under which circumstances premature beats become a clinical concern for the horse are questions which therefore remain. Consequently, large-scale frequency studies of equine arrhythmias – standardized in terms of recording duration, exercise intensity, and definitions of arrhythmia – are required.

Many arrhythmias are paroxysmal, and comprehensive evaluation of their frequency therefore requires long-term ECG recording for 24 h or more [14]. This makes ECG analysis very time-consuming and would constitute a substantial obstacle in conducting large-scale studies of equine arrhythmias. In human ECG studies, analysis of long-term recordings is commonly facilitated by automatic detection and classification of arrhythmias based on timing and morphology of ECG complexes [15]. However, no morphology algorithms are available for equine ECG analysis, as these would be impeded by the highly variable equine T-wave morphology. Computerized methods for the analysis of equine ECGs are therefore limited to timing algorithms, identifying beats with abnormal timing by assessing the variation in beat-to-beat intervals against an observer-specified threshold. These timing algorithms are, however, only an aid to the observer, who must still manually classify the arrhythmia category of every rhythm event detected by the algorithm, as well as manually assess the morphology of ECG complexes to identify any possible ectopic beats with normal timing. Nevertheless, with approximately 50,000–60,000 heartbeats in a 24-h ECG recording, timing algorithms represent an invaluable time-saving tool.

The aim of this study was to provide suggestions for a standardized approach to semi-automatic assessment of large equine ECG datasets. In order to achieve this, the following objectives were set: 1) to investigate the beat-to-beat variation of equine cardiac rhythm in order to determine appropriate threshold levels of maximum acceptable RR deviation, and 2) to evaluate a novel two-step timing algorithm for equine ECG analysis by quantifying the frequency of arrhythmias in a cohort of clinically healthy endurance horses both at rest for 24 h, as well as during moderate exercise training.

## Methods

### Horses

Eleven Arabian breed endurance horses were included in the study: 1 mare, 2 stallions and 8 geldings, with a median age of 11 years (range 8–15 years), and a median body weight of 415 kg (range 359–503 kg). A twelfth horse was excluded (see Results). All horses were in full training at the time of examination and successfully completed an endurance ride ≥80 km within 38 days of the examination (median, range 5–105 days). All horses were considered healthy, based on the performance history obtained from the owner and a clinical examination. A cardiac murmur exceeding grade 2/6 was considered an exclusion criterion [16]. Additionally, standardized echocardiography was used to rule out cardiac pathology [5]. The study conformed to the guidelines for non-invasive ethical animal research as dictated by Danish law (The Animal Experimentation Act 1306 of 23rd Nov 2007). Due to the non-invasive characteristics of the protocol, no blood samples were collected. Written informed consent was obtained from the horse owners.

### ECG recordings

All ECG recordings were obtained with a Holter recording system at a sampling frequency of 500Hz.^1^ Electrodes were placed in a modified base-apex lead and secured by adhesive foam pads.^2^ Two bipolar leads (lead I and II) were recorded by two separate channels and a third lead (lead III) was computed by the Holter system. The ground electrode and the combined negative electrode of leads I and II were placed dorsally over the region of the left scapula. The positive electrode of lead I was placed in the left flank in the middle of the most caudal rib, and the positive electrode of lead II was placed caudomedial to the pectoral muscles and to the left of the ventral midline. Resting ECGs were obtained over a period of 24 h when the horses were confined to their stall or a small paddock. Exercise ECGs were obtained during a regular training session. The exercise protocol included six intervals of increasing velocities: one at walk, two in trot, two in canter and one in gallop. Each interval lasted for 3–10 min, or the equivalent of approximately 3,000 m. This ensured that a wide range of heart rates (HR) were included in the ECG recordings during exercise. The recordings were stored digitally and transferred to a computer using the software provided with the recorder.^1^ All ECG recordings were manually reviewed in the first instance to assess the recording quality and the morphology of QRS complexes, so as to detect possible ventricular ectopic beats with normal timing that could potentially be “overlooked” by the timing algorithm later in the process. The RR intervals and associated time points were subsequently exported for further analyses in SAS.^3^^,^ 4 Sequences with excessive noise and ECG artifact, caused for example by electrode detachment, were excluded separately in SAS, and exclusion of >10% of the recording duration resulted in exclusion of the entire recording. The heart rate was calculated as a moving average of 10 beats (arrhythmic beats excluded), and the mean, minimum and maximum heart rate of every hour of the 24-h ECG recording at rest was assessed. In addition, the following HRV time domain measures were calculated from the 24-h ECG recordings at rest: SDNN = standard deviation of NN (normal RR intervals) calculated over the entire 24-h period, SDANN = standard deviation of the means of NN intervals calculated over all 5-min sequences during the 24-h period, SDNN index = the mean of all 5-min standard deviations of NN, and pNN50 = proportion of successive NN intervals that differed more than 50 ms.

### Cardiac beat-to-beat variation

For each individual horse, the percentage RR deviation (RR_dev(i)_ = (RR_i_-RR_i-1_)/RR_i-1_*100%) was plotted against the RR interval (RR_i_) to display the beat-to-beat variation. This RR deviation plot is a modification of a Poincaré plot (RR_i+1_ against RR_i_) and therefore results in a main cluster of sinus beats with normal timing around the line of identity [17], which in the case of the RR deviation plot is represented by the horizontal axis (RR_dev_ = 0). The margins of this main cluster were then visually assessed for each individual horse and plotted together in one superimposed plot to allow for comparison of the beat-to-beat variation among horses. Based on this superimposed plot, a general threshold model with three different HR ranges was defined: “*Rest*” HR <60 bpm; “*Transition*” HR = 60–100 bpm, and “*Exercise*” >100 bpm. According to the definition of RR deviation, intervals longer than the preceding interval (RR_i_ > RR_i-1_) resulted in positive values of RR_dev_, while shorter intervals (RR_i_ < RR_i-1_) produced negative values of RR_dev_. Premature beats would therefore obtain negative values of RR_dev_, and the threshold level of maximum acceptable RR deviation (RR_max,dev_) in each HR range was consequently determined by the largest negative RR deviation observed among the horses.

### Two-step timing algorithm

**Fig. 1:**
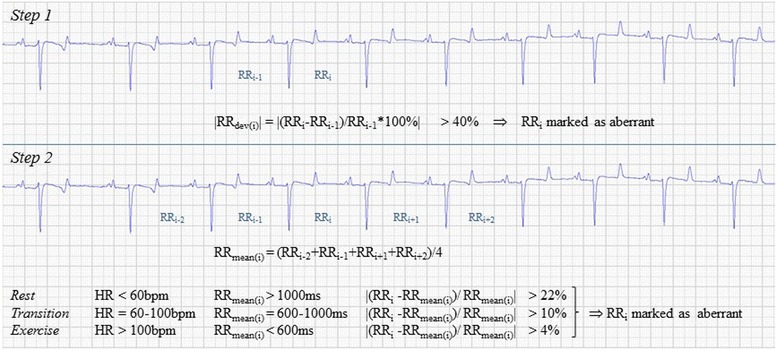
Schematic presentation of the two-step algorithm

A two-step timing algorithm incorporating the defined threshold model was then elaborated to identify beats with aberrant timing that were suspected of being arrhythmic (Fig. 1):

#### Step 1

This step served as a preliminary sorting where the beats with the most clearly aberrant timing (characterized by a |RR_dev_| >40%) were marked as “aberrant”. These aberrant RR intervals were temporarily excluded and the remaining RR intervals linked together to create a temporary data series. This prevented excessively long intervals (i.e. blocks and pauses) from interfering with the calculation of a local mean in the next step.

#### Step 2

A modified Ho-Goldberger algorithm [18] was then run on the temporary data series to point out *additional* aberrant intervals. This algorithm compared each individual interval (RR_i_) to a local mean of the four most proximal intervals (RR_mean(i)_ = (RR_i-2_ + RR_i-1_ + RR_i+1_RR_i+2_)/4) against the threshold level of RR_max,dev_. The relevant HR range in the threshold model was determined by RR_mean(i)_ as an indicator of the underlying HR in the sequence.

### Classification of arrhythmias

In order to evaluate the performance of the two-step timing algorithm, all RR intervals marked as “aberrant” were traced back to the ECG recordings for manual classification according to the following definitions:

- *Sinoatrial block:* a pause in the cardiac rhythm where the PP interval is equal to a multiple of the baseline PP interval [19].
- *Sinus arrest:* a pause in the cardiac rhythm where the PP interval is *not* a multiple of the baseline PP interval and is more than twice the baseline PP interval (i.e. longer than a sinoatrial block) [19].
- *Delayed sinus beat:* a pause in the cardiac rhythm that is less than double the baseline PP interval (i.e. shorter than a sinoatrial block).
- *Sinus arrhythmia:* an irregularity of the cardiac rhythm (beyond what can be expected as normal beat-to-beat variation) with variable PP intervals that might show a cyclic pattern [19].
- *Wandering pacemaker:* an alternating P-wave morphology as the pacemaker function shifts between the sinus node and various ectopic foci in the atria [20].
- *2nd degree AV block:* the P wave is not succeeded by a QRS complex, as the electrical impulse is blocked in the AV node [19].
- *Supraventricular premature complex (SVPC):* an ectopic beat of atrial origin occurring too early (premature) according to the threshold of beat-to-beat variation [21]. The P-wave morphology may be changed depending on the location of the ectopic pacemaker. The SVPC might be followed by a *compensatory pause* if the ectopic impulse fails to enter and reset the sinus node [19].
- *Ventricular premature complex (VPC):* an ectopic beat from the ventricular myocardium occurring too early according to the threshold of beat-to-beat variation. The QRS complex is not related to a preceding P-wave and its morphology is often wide and bizarre, depending on the location of the ectopic pacemaker [22]. The VPC is often followed by a *compensatory pause*, but depending on the timing of the ectopic impulse, the VPC may also become *interpolated* between two sinus beats [19]. A special VPC variant is a *fusion beat* which occurs simultaneously with a sinus beat (not premature) [19].
- *False aberrant:* a normal RR interval in sinus rhythm incorrectly marked as aberrant by the two-step algorithm (e.g. due to neighboring arrhythmias).
- *Technical errors:* collective classification of aberrant RR intervals caused by QRS undersensing or ECG artifact.

### Statistical analysis

The arrhythmia frequencies of the individual horses were calculated separately for rest and exercise ECG recordings. Values are given as median and range.

## Results

**Fig. 2:**
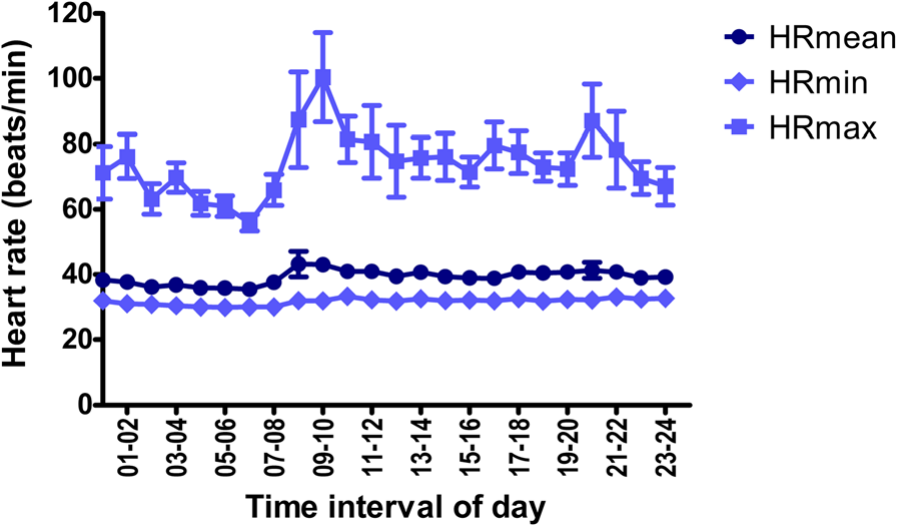
The hourly heart rate during the 24-h EKG at rest. HRmean; average heart rate. HRmin; minimum heart rate. HRmax; maximum heart rate. Values presented as mean ± SEM

One exercise ECG and one resting ECG (from different horses) were excluded from the arrhythmia classification as they were not considered to be of diagnostic quality. A twelfth horse was initially included in the study population, but showed excessive numbers of sinoatrial blocks and sinus arrests of up to 9 s long throughout the 24-h recording at rest. Although the condition resolved during exercise, this horse was suspected of sinus node dysfunction and excluded from the study. The hourly mean, minimum and maximum HR in the ECG recordings at rest is presented in Fig. 2. The HRV time domain measures were as follows (mean ± SD): SDNN was 233 ± 40 ms, SDANN was 160 ± 37 ms, SDNN index was 144 ± 30 ms, and pNN50 was 33 ± 12%. The maximum HR averaged over 10 beats during exercise was 188 bpm (156–209 bpm). Echocardiography revealed trivial aortic regurgitation in three horses, but no other valvular insufficiencies or abnormal cardiac dimensions were observed.

### Threshold levels of beat-to-beat variation

**Fig. 3:**
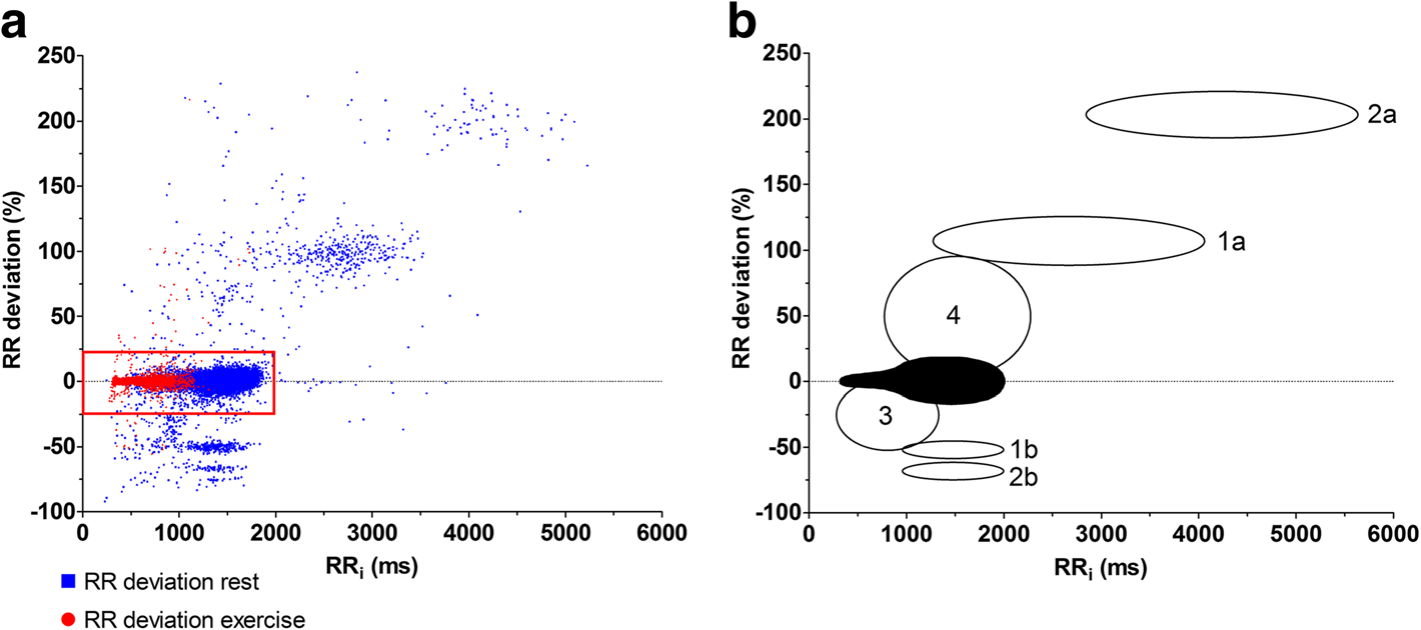
RR deviation plot (**a**) and schematic presentation of beat patterning (**b**). Notice the main cluster (*red square*) surrounded by smaller clusters. The black area represents the main cluster of normal sinus beats. Area 1a represents sinoatrial blocks, second-degree AV blocks and QRS undersensing with double the length of normal RR intervals and RR_dev_ ≈ 100%. Similarly, area 2a describes QRS undersensing of two consecutive complexes. Areas 1b and 2b represent normal RR intervals succeeding the aberrant intervals of areas 1a and 2a and are therefore located below the main cluster with an RR_dev_ of approximately -50% and -66%, respectively. Area 3 refers to premature beats (both supraventricular and ventricular) while area 4 refers to *“Delayed sinus beats”*. Aberrant intervals caused by artifact were scattered throughout the plot

**Fig. 4:**
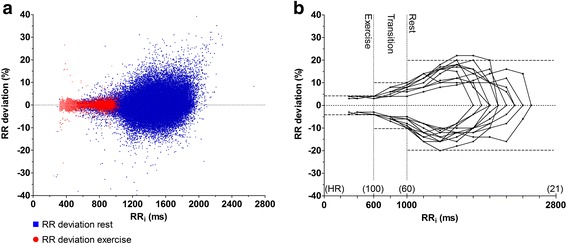
Zoom in on the main cluster in the RR deviation plot of a representative horse (**a**). The club-shape clearly illustrates that RR intervals at rest (*blue*) show larger RR deviation (RR_dev_) than RR intervals during exercise (*red*). Notice the slight asymmetry with a larger dispersion of positive values of RR_dev_ at rest. The visually extrapolated margins of the main cluster of individual horses were superimposed for direct comparison (**b**). The dashed lines indicate heart rate ranges and threshold levels of maximum acceptable RR deviation. Note the consistent pattern of small RR_dev_ with little inter-horse variation during exercise, gradual increase of RR_dev_ in the transition from exercise to rest, and large inter-horse variation at rest

The RR deviation plots showed a distinct pattern with a “club-shaped” main cluster surrounded by smaller clusters. Retracing the beats in different clusters to the ECG confirmed that the main cluster represented normal beats in sinus rhythm, while surrounding clusters revealed a consistent beat pattern associated with the different arrhythmia categories (Fig. 3). The club-shaped main cluster had its narrowest part (“the handle”) at shorter RR intervals and was gradually enlarged with increasing RR intervals. Additionally, it was slightly asymmetric around the horizontal axis (RR_dev_ = 0) with wider dispersion of data points above the axis (Fig. 4). At RR intervals <600 ms (HR >100 bpm), the margins of the main cluster representing the normal RR_dev_ ranged from 3% to 4% in all horses. With intervals of 600-1000 ms (HR = 60–100 bpm), RR_dev_ gradually increased and ranged between 4% and 10% with the largest RR_dev_ at lower HRs. At RR intervals >1000 ms (HR <60 bpm), RR_dev_ showed considerable inter-horse variation and slight asymmetry with peak positive values ranging from 12% to 22% and peak negative values from 10% to 20%. Due to the apparent association between the beat-to-beat variation and HR, three different HR ranges (“Rest” <60 bpm; “Transition” = 60–100 bpm, and “Exercise” >100 bpm) were included in the threshold model. The threshold levels (RR_max,dev_) of each HR range were defined based on the cluster margins described above and were evaluated by a trial-and-error approach, where the threshold value was adjusted multiple times and beats with an RR deviation just below and above the threshold were assessed. The final threshold values were then defined based on these observations and set at 20%, 10%, and 4%, respectively (Fig. 4b).

### Arrhythmias

**Fig. 5:**
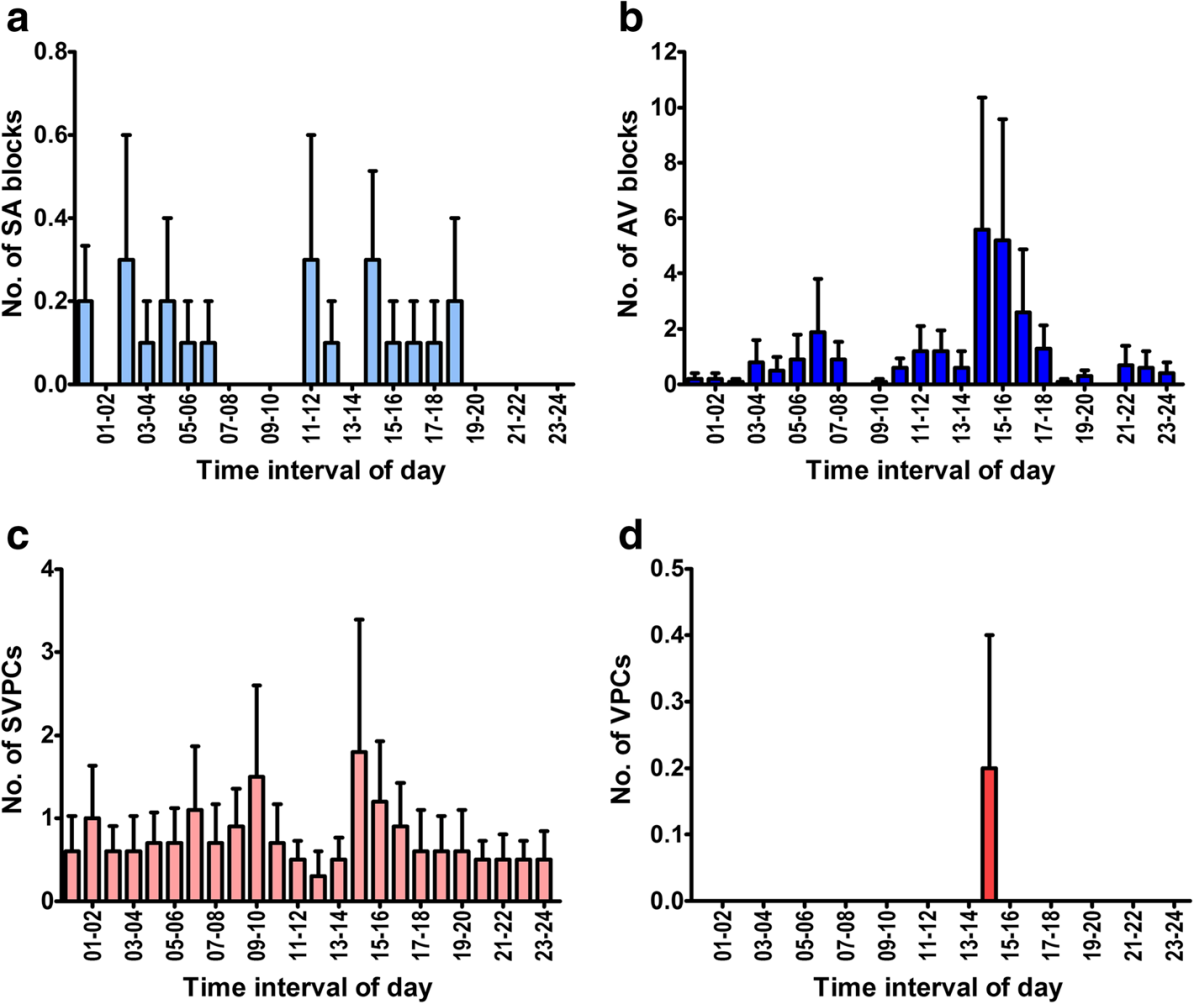
The hourly frequency of arrhythmias during the 24-h ECG recordings at rest. **a** Sinoatrial (SA) blocks. **b** Second degree atrioventricular (AV) blocks. **c** Supraventricular premature complexes (SVPC). **d** Ventricular premature complexes (VPC). Bars indicate mean ± SEM. Note the different scales

**Fig. 6:**
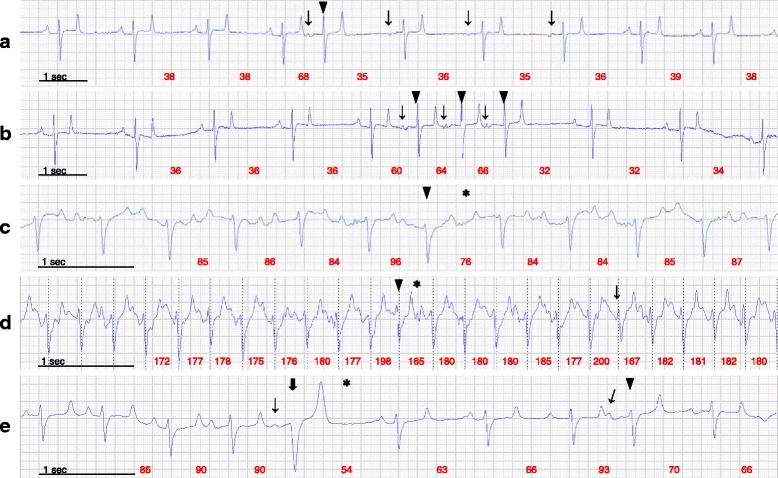
Premature beats. **a** SVPC (arrowhead) without compensatory pause occurring at rest. Note the transient change in P-wave morphology (arrows). **b** Triple SVPC occurring at rest. Note the abnormal P-wave morphology (arrows). **c** and **d** SVPC (arrowhead) followed by a fully compensatory pause (*) occurring during walk and gallop, respectively. Note the presence of movement artifact on the baseline obscuring the P-wave, and the technical error in triggering the peak R-wave (arrow). **e**) VPC possibly a fusion beat (thick arrow) followed by a compensatory pause (*) and an SVPC (arrowhead) at rest. An abnormal and possibly unconducted P-wave (arrow) precedes the VPC. Note the varying P-wave morphology. The instantaneous heart rate is shown in red

**Fig. 7:**
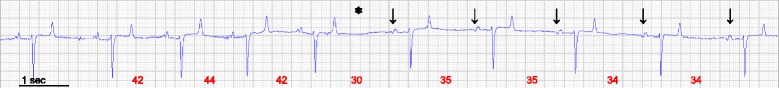
Delayed sinus beat (*). This beat coincided with a change of pacemaker, indicated by altered P-wave morphology (arrows) in one horse with wandering pacemaker. Note the decrease in instantaneous heart rate (*red* numbers)

Aberrant intervals marked by the two-step algorithm constituted up to 1.7% of the total number of beats at rest and 3.8% during exercise. Manual classification of these intervals revealed that actual arrhythmias constituted up to 0.4% of the total beats in both the rest and exercise recordings (Table 1). The remaining aberrant intervals were: *delayed sinus beats*; *false aberrants* and *technical errors*. The hourly frequencies of arrhythmias during the 24-h ECG recordings at rest are displayed in Fig. 5. Seven horses had SVPCs in varying numbers (median 7, range 2–86) at rest, and six horses had SVPCs (median 2, range 1–24) during exercise (Fig. 6a–d). The two-step algorithm detected two VPCs (Fig. 6e), and no additional VPCs were detected during the initial manual review. A changed P-wave morphology indicating ectopy could be confirmed in 52% of the SVPCs at rest, and in 6% of the SVPCs during exercise. For the remaining SVPCs, the timing of the beat was the sole indicator of the arrhythmia. Varying P-wave morphology (Fig. 6e) suggesting a wandering pacemaker was observed in five horses, and pacemaker “shifts” occasionally coincided with SVPCs (Fig. 6a) or *“Delayed sinus beats”* (Fig. 7).

**Table 1:** Frequency distribution of arrhythmias during rest and exercise

			Rest						Exercise	
Horse	Age (years)	Sex	Recording duration (hours)	Sinoatrial block	Sinus arrest	2nd degree AV block	SVPC	VPC	Recording duration (hours)	SVPC
1	11	G	24.0	1	0	0	0	0	1.6	0
2	14	G	23.3	0	0	0	6	0	1.6	1
3	9	G	22.9	0	0	211	7	0	N/A	N/A
4	15	S	N/A	N/A	N/A	N/A	N/A	N/A	1.3	5
5	8	G	24.0	0	0	1	0	0	1.4	1
6	8	G	24.0	7	0	3	4	0	1.8	2
7	15	G	24.0	0	0	0	86^a^	2	1.6	24
8	8	S	22.1	0	0	0	2	0	1.6	0
9	13	G	24.0	13	1	42	69	0	1.4	1
10	9	M	24.0	1	0	3	7	0	1.5	0
11	11	G	24.0	0	0	0	0	0	1.7	0

*G* gelding, *M* mare, *S* stallion. *SVPC* supraventricular premature complex, *VPC* ventricular premature complex. *N/A* not available. ^a^Including three episodes of double SVPCs and one triple SVPC

## Discussion

This study is the first to provide recommendations on threshold levels of beat-to-beat variation for equine ECG analysis, and presents an adaptive model where threshold levels (RR_max,dev_) are adjusted according to HR range. RR deviation is essentially a measure of heart rate variability (HRV), and in contrast to conventional measures of HRV such as SDNN (standard deviation of normal RR intervals), knowledge of the beat-to-beat RR_dev_ can be applied directly to the ECG analysis. The observation that beat-to-beat variation is larger at rest than during exercise agrees with existing knowledge that parasympathetic activity is responsible for equine HRs below 110–130 bpm, and that HRV is positively correlated with parasympathetic activity [23–26].

An interesting observation in the RR_dev_ plot was the asymmetric appearance of the main cluster with wider dispersion of positive values of RR_dev_ at rest (Fig. 4). This asymmetric distribution of RR_dev_ indicates a non-linear component in the beat-to-beat variation of equine cardiac rhythm, where the impulse formation in the sinus node is more tightly regulated with regards to beats that appear “early” than beats that occur “delayed” (cf. the definition of RR_dev_). Nonlinearity is a well-known trait of human HRV [27], and is rooted in the sympathetic/parasympathetic regulation of cardiac rhythm. In humans, short-term heart rate asymmetry has been shown to be dominated by heart rate decelerations (i.e. prolongations of the RR interval), whereas long-term and total heart rate asymmetry are primarily contributed by accelerations (i.e. RR interval shortenings) [28]. The exact causal mechanisms of heart rate asymmetry are unknown, but it is assumed be regulated by similar factor as HRV. These factors include the baroreceptor reflex and respiratory sinus rhythm modulations, but heart rate asymmetry has furthermore been linked to asymmetry in the myocardial conduction time between the atria and ventricles [29]. It remains unclear how heart rate asymmetry is modulated in horses. However, taking the dominant vagal tone of this species into consideration and the fact that the asymmetry in the RR_dev_ plot was only observed at resting hearts, it could be expected that heart rate deceleration (RR interval prolongations) might be the dominant contributor of both short-term and long-term heart rate asymmetry in horses – although this remains speculations. We chose to define the threshold levels based on the maximum negative peak beat-to-beat variation observed among the horses, in order to optimize the detection of premature beats. However, since the negative peak values of beat-to-beat variation were lower than positive peak values, a number of normal sinus beats with elongated RR intervals (delayed sinus beats) were consequently marked as aberrant. For this reason, traditional timing algorithms were rejected in favor of the Ho-Goldberger algorithm [18], which allows more physiological variation between two consecutive intervals than specified by RR_max,dev_, providing the deviation from the local mean does not exceed the threshold. It is therefore less sensitive to minor physiologic rhythm variations such as sinus arrhythmia and delayed sinus beats*,* than traditional algorithms, where each RR interval (RR_i_) is compared with only the preceding interval (RR_i-1_). Nevertheless, timing algorithms merely identify beats with aberrant timing that are suspected of being arrhythmic, and the observer must still assess whether the “aberrant” beat is truly arrhythmic or simply caused by physiologic beat-to-beat variation. A higher level of accuracy in the threshold levels used will result in a more reliable timing algorithm. The algorithm presented in this study was very sensitive, and a number of the RR intervals marked as aberrant were not caused by actual arrhythmias, but instead by ECG artifacts, QRS undersensing or physiologic sinus arrhythmia (these beats were classified as technical errors, false aberrant or delayed sinus beat). However, ectopic beats could potentially go unnoticed should they occur within the “normal timing” defined by the RR_max,dev_ (as is the case for *fusion beats*). Ideally, the observer should therefore evaluate the morphology of every single complex to check for undetected ectopic beats. This is relatively straightforward with beats of ventricular origin since they commonly have an unusual appearance (with the exception of those originating from the AV nodal area – i.e. *junctional beats* [22]). However, validation of the number of detected SVPCs is more complicated since the ectopic nature of these beats often cannot be confirmed by altered P-wave morphology. Without verifiable changes of the P-wave, it can be difficult to distinguish SVPCs from physiologic rhythm variations. Conversely, in horses with wandering pacemaker, a change of pacemaker can be associated with a shift in impulse rate and thereby mimic an SVPC (Fig. 6a). A number of beats classified as SVPCs in this study with obscured P waves were followed by a compensatory pause, and it is possible that some of these beats might actually be of ventricular origin, despite the normal appearance of the QRS complex. Other authors have refrained from classifying such “*isolated premature cycles*” as either supraventricular or ventricular which might be a more appropriate approach [7]. This again illustrates the need for more standardized analysis criteria in the assessment of equine ECGs.

Although a predisposition to arrhythmias has been demonstrated in human endurance athletes [30, 31], no studies have evaluated the occurrence of arrhythmias in endurance horses. The limited number of horses examined in this study is not sufficient to provide prevalence or frequency estimates for the general population. However, in agreement with previous studies in other types of horses [4–6, 8], the results suggest that SVPCs are relatively common in endurance horses. As stated in the Introduction, the appropriate clinical question is therefore related more to the *frequency* of premature beats in a given time period or under specific circumstances (e.g. exercise) than to their *prevalence*. However, the existing diverse study designs with inconsistent or inadequate definitions of premature timing prevent general conclusions on this matter. One human study has suggested a “normal range” for both SVPCs and VPCs of up to 200 per 24 h [32], though considering the low frequency of premature beats observed in this study, extrapolating this suggestion to horses is questionable. Large-scale frequency studies of arrhythmias in horses are therefore required.

The study presented here should be considered as a first practical step in defining suitable threshold values of normal RR deviation in equine ECG analysis. The cohort of horses examined in this study is small, and as such, it cannot be the basis of standards for the horse population in general. A number of limitations should therefore be addressed in future studies, and further investigations will require a larger number of animals in order to determine if the results can be extrapolated to larger populations and other breeds of horses. Ideally, future studies should also include a separate population for validation of the results. The ECG recording system used in this study is limited to three ECG leads. The P-wave morphology of an ectopic beat will therefore not necessarily appear abnormal on the surface ECG. Using more ECG leads from different angles could improve depiction of the P wave and thereby facilitate the classification of arrhythmias. Finally, it would be beneficial to include information on electrolyte levels and cardiac markers in the clinical evaluation of the horses.

## Conclusions

This study presents a novel approach to semi-automatic ECG analysis in horses, with recommendations on threshold levels of beat-to-beat variation and a novel two-step timing algorithm which takes the physiological rhythm variation into account. Furthermore, the study provides preliminary results on arrhythmia frequencies in endurance horses during both rest and moderate exercise. The presented approach should be validated in a larger population of horses, but could be beneficial in future large-scale frequency studies which aim to determine the clinical significance of various arrhythmias in horses.

## Abbreviations

Bpm: Beats per minute
HR: Heart rate
HRV: Heart rate variability
RR_dev_: Deviation of RR intervals
RR_max,dev_: Maximum acceptable deviation of RR intervals

## Footnotes

1 Televet 100, Engel Engineering Services GmbH, Heusenstamm, Germany

2 Animal Polster, Snøgg AS, Kristiansand, Norway

3 Microsoft Excel 2010, Microsoft Corp, Redmond, WA, USA

4 SAS Enterprise Guide 6.1, SAS Institute, Cary, NC, USA
